# A Mobile Phone–Based Sexual and Reproductive Health Intervention for Female Sex Workers in Kenya: Development and Qualitative Study

**DOI:** 10.2196/15096

**Published:** 2020-05-29

**Authors:** Frances H Ampt, Kelly L'Engle, Megan S C Lim, Kate F Plourde, Emily Mangone, Collins Mudogo Mukanya, Peter Gichangi, Griffins Manguro, Margaret Hellard, Mark Stoové, Matthew F Chersich, Walter Jaoko, Paul A Agius, Marleen Temmerman, Winnie Wangari, Stanley Luchters

**Affiliations:** 1 Burnet Institute Melbourne Australia; 2 Department of Epidemiology and Preventive Medicine Monash University Melbourne Australia; 3 University of San Francisco San Francisco, CA United States; 4 FHI 360 Durham, NC United States; 5 School of Nursing University of North Carolina at Chapel Hill Chapel Hill, NC United States; 6 mHealth Kenya Nairobi Kenya; 7 Technical University of Mombasa Mombasa Kenya; 8 Department of Public Health and Primary Care International Centre for Reproductive Health Ghent University Ghent Belgium; 9 International Centre for Reproductive Health Mombasa Kenya; 10 Department of Infectious Diseases The Alfred Hospital Melbourne Australia; 11 Wits Reproductive Health and HIV Institute University of the Witwatersrand Johannesburg South Africa; 12 University of Nairobi Nairobi Kenya; 13 Aga Khan University Nairobi Kenya

**Keywords:** sex work, mobile health (mHealth), unintended pregnancy, qualitative research

## Abstract

**Background:**

Female sex workers (FSWs) have high rates of both unintended pregnancy and HIV, but few health promotion interventions address their contraceptive needs or other sexual and reproductive health and rights (SRHR) concerns. A broader approach integrates contraceptive promotion with HIV and sexually transmitted infection (STI) prevention and management, alcohol awareness, gender-based violence and rights, and health care utilization. The Women’s Health Intervention using SMS for Preventing Pregnancy (WHISPER) mobile phone intervention uses a participatory development approach and behavior change theory to address these high-priority concerns of FSWs in Mombasa, Kenya.

**Objective:**

This paper aimed to (1) describe the process of development of the WHISPER intervention, its theoretical framework, key content domains and strategies and (2) explore workshop participants’ responses to the proposed intervention, particularly with regard to message content, behavior change constructs, and feasibility and acceptability.

**Methods:**

The research team worked closely with FSWs in two phases of intervention development. First, we drafted content for three different types of messages based on a review of the literature and behavior change theories. Second, we piloted the intervention by conducting six workshops with 42 FSWs to test and refine message content and 12 interviews to assess the technical performance of the intervention. Workshop data were thematically analyzed using a mixed deductive and inductive approach.

**Results:**

The intervention framework specified six SRHR domains that were viewed as highly relevant by FSWs. Reactions to intervention content revealed that social cognitive strategies to improve knowledge, outcome expectations, skills, and self-efficacy resonated well with workshop participants. Participants found the content empowering, and most said they would share the messages with others. The refined intervention was a 12-month SMS program consisting of informational and motivational messages, role model stories portraying behavior change among FSWs, and on-demand contraceptive information.

**Conclusions:**

Our results highlight the need for health promotion interventions that incorporate broader components of SRHR, not only HIV prevention. Using a theory-based, participatory approach, we developed a digital health intervention that reflects the complex reality of FSWs’ lives and provides a feasible, acceptable approach for addressing SRHR concerns and needs. FSWs may benefit from health promotion interventions that provide relevant, actionable, and engaging content to support behavior change.

## Introduction

HIV prevention programs for female sex workers (FSWs) utilizing peer educators, drop-in-centers, and mobile outreach have been implemented in sub-Saharan Africa [1,2] and have shown promise in improving condom use, HIV and sexually transmitted infection (STI) prevalence, and HIV testing [3-5]. However, the broader sexual and reproductive health and rights (SRHR) needs of this population have been largely neglected by a narrow focus on HIV [6], potentially limiting the effectiveness of prevention programs [7] and prompting calls for greater integration of family planning, community empowerment, gender-based violence, and antenatal care services into existing programs [1,8-12].

Pregnancy prevention is a particular area of need for FSWs, with high rates of unintended pregnancy and low uptake of highly effective contraception and dual method use among those wanting to avoid pregnancy [13,14]. Research with FSWs in Mombasa, a port city and transport hub on Kenya’s East Coast with a large FSW population [15], documented that over 1 year, 24% had an unintended pregnancy and only 57% were using a modern contraceptive method [16].

Limited knowledge of long-acting reversible contraceptives (LARCs), fear of side effects, and social and gender norms that limit the use of family planning are common among FSWs in this setting [11,16-19] and women in sub-Saharan Africa more generally [20,21]. This indicates a critical need for messaging that addresses family planning knowledge, attitudes, and behaviors in the context of sex work. Mobile phones offer a promising medium for such communication, as they are increasingly used to arrange sex work encounters and solicit clients [22], can reach marginalized populations with low engagement in formal services, and mobile coverage is high in most countries (eg, 96% in Kenya) [23].

Mobile phones have been used to deliver health promotion in a variety of contexts, and this approach has been effective in improving knowledge, use, and continuation of contraception [24], as well as impacting preventive behaviors for other health domains [25]. Mobile health (mHealth) interventions have not been implemented with FSWs, but they have been evaluated with young people and postpartum women in sub-Saharan Africa, and have successfully impacted contraceptive outcomes in these contexts [26-28].

We developed a mobile phone intervention for FSWs in Mombasa to promote contraceptive use—particularly LARCs—and other behaviors related to SRHR. This intervention, called the Women’s Health Intervention using SMS for Preventing Pregnancy (WHISPER), is being tested in a cluster-randomized controlled trial (RCT) to assess its impact on unintended pregnancy [29].

The intervention was developed using a participatory design approach. FSWs in Mombasa were involved in the initial conception of the intervention and in formal workshopping and testing. Participation by the target community in intervention design [30] and the development of health programs [31] may lead to greater health impacts. However, participatory design methods for mHealth interventions with minority populations such as FSWs are rarely explicitly described [32].

In this paper, we aim to (1) describe the development of the WHISPER intervention and present its theoretical framework, key content domains, and strategies, and (2) explore workshop participants’ responses to the proposed intervention, particularly with regard to message content and behavior change constructs. Finally, we present the schedule and approach for intervention implementation and delivery.

## Methods

### Summary

Methods for the development of WHISPER have been described by Ampt et al [29] and generally follow the steps outlined by L’Engle et al [33]. The intervention was developed in two phases: first, to design the intervention framework and draft content; and second, to pilot the intervention with FSWs, refine the messages based on the results, and finalize the intervention structure and content.

### Phase 1: Developing the Framework and Draft Content

The framework for intervention content, and the drafting of initial messages, was informed by the following: review of the literature on motivators and barriers to FSWs’ adoption of healthy SRHR behaviors; consideration of health promotion theory, specifically transtheoretical [34] and social cognitive [35] theories; and consultation with FSWs who formed part of the research team. These women were experienced peer educators at the International Center for Reproductive Health’s drop-in centers and came from the targeted FSW communities. We incorporated messaging from existing mHealth repositories and previous programs developed by the investigators [36-38] and aligned the content with relevant Kenyan and global guidelines for family planning [39,40] tailored to the specific needs of sex workers including their high risk of STIs and HIV [41]. We drafted and tested the messages in English rather than Kiswahili following advice from the Kenyan research team and peer educators.

#### Intervention Framework

A review of the literature and behavior change theory highlighted key content domains and corresponding behavioral factors that impact the risk of unintended pregnancy, STIs, and HIV.

**Figure 1:**
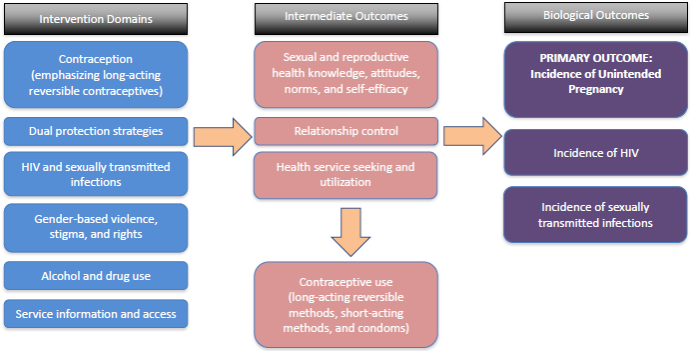
The Women’s Health Intervention using SMS for Preventing Pregnancy program logic.

These domains and factors were confirmed as important and relevant to FSWs during consultations with peer educators and were incorporated into a logic model (Figure 1) that guided content development. The peer educators agreed that pregnancy prevention was a high priority and also identified conflicting attitudes to family planning in the community, due to fear of side effects and myths about the effects of some methods, particularly intrauterine devices (IUDs). The use of condoms for STI and HIV prevention was recognized as important, but a number of barriers to correct and consistent use were identified. Violence from clients and other partners, as well as heavy alcohol use, were also highlighted.

#### Intervention Strategies

The intervention was designed to incorporate specific cognitive strategies from behavior change communication theory [42,43] and appeal to women at different stages of change [34] (Table 1). Three different types of messages were used: discrete messages of less than 160 characters *pushed* to participants’ phones on a predetermined schedule; role model stories, consisting of narratives about FSWs negotiating SRH risks, sent to participants’ phones over several messages (episodes); and on-demand (*pull*) messages that participants could access at any time by replying to messages with specific codes.

#### Push Messages

These messages provided specific information in less than 160 characters (1 standard SMS) and used strategies to motivate and educate participants. In the precontemplative stage, more of the messages aimed at the women were developed to be sent early in the intervention, with greater emphasis on action and maintenance later on. However, there was a mixture throughout, given the anticipated diversity of stages of change of participants. The sequencing of role model stories also reflected this approach. Push messages were delivered on alternating months to role model stories. Examples of push messages and their associated behavior change strategies are provided in Table 1.

**Table 1:** Example messages and a role model story episode mapped to behavior change theory and strategies.

Intervention domain		Example message^a^		Stage of change and definition		Cognitive strategies	
**Stand-alone** **push** **messages**							
	Contraception		We have something important to tell you. Family planning lets you have sex without getting pregnant. That’s what WHISPER is all about.		Precontemplation: not yet thinking about changing behavior		Increase awareness of risk; set positive outcome expectations; attract attention, *brand* recognition (social marketing strategy); frame subsequent messages
	Dual protection		Husband or boronga (type of client)? (No matter who they are, they should be wearing a condom if they want to be with you). Hugs and kisses from WHISPER.		Precontemplation		Improve knowledge; use a friendly and personal tone to provide positive encouragement and social support; use humor to highlight desired behavior
	Contraception		Most women who use family planning continue to have a normal sex drive. If you find one method leaves you without a sexual appetite, there are many other options.		Contemplation and preparation: thinking about making changes in behavior		Improve knowledge; challenge outcome expectations (related to fears of side effects); address specific concerns; provide an alternative strategy
	HIV and STIs		Did you know you can take a rapid test for HIV? You get the result straight away, so you don’t have to come back later! Reply 100 for services that do testing.		Contemplation and preparation		Set positive outcome expectations; motivate; provide specific action strategy
	Gender-based violence, stigma, and rights		Violence against women is not ok, and it’s not your fault. If you experience violence, remember you are not alone and can get help. Hugs, WHISPER.		Contemplation and preparation		Change social norms and model empowerment; provide social support; build self-efficacy for getting help; encourage help-seeking behavior
	Alcohol and drug use		You can reduce your drinking: ask for beer bottles filled with water, add water to mixed drinks, secretly dump some out, drink soda, drink slow. WHISPER.		Preparation and action: preparing to act or taking actions to change behavior		Build skills and self-efficacy by breaking down behavior into components; develop action plans; encourage goal setting
	Service information and access		If you have a bad experience with a health care provider, don’t give up—ask your peer educator for clinic recommendations. Kisses and hugs.		Action and maintenance: taking actions to change behavior, for 6 months or more (maintenance)		Improve self-efficacy by overcoming setbacks; build skills to prevent or address relapse; provide alternative strategies
**Role model story (episode 1)**							
	Contraception		Karibu tujienjoy [Welcome, let's have fun]! I’m Ciku from WHISPER. I’m new to town: I left my village because my husband drank a lot and was violent. I might be young, but I know I deserve better. I have some mpenzi [lovers] who help me out but I’ve had a couple of scares at the clinic, if you know what I mean. I need a better way to prevent pregnancy!		The character moving from precontemplation to contemplation in this episode.		Personalize, set scene; model self-efficacy and empowerment (leaving a violent relationship); present negative outcome expectations (risks of current behavior)

^a^Example messages contain final content, including any modifications made during phase 2.

#### Role Model Stories

Role modeling healthy behaviors through stories about relatable peers constitute a recognized social-cognitive strategy for behavior change [42] but have rarely been used in mHealth. The WHISPER role model stories were intended to be delivered as multiple episodes, describing FSWs who overcome barriers to contraceptive use by modeling healthy social norms and behaviors. To develop the stories, peer educators workshopped common and engaging scenarios that highlighted FSWs’ risk of pregnancy (Table 2). The research team used these as the basis for developing and testing six stories, each promoting the use of LARCs integrated with other relevant themes. Peer educators also provided ideas about character names, language, and narrative, which were incorporated into the stories to ensure their relevance to the FSW community.

**Table 2:** Role model stories developed from peer educator consultation.

Scenarios from FSW^a^ peer educators	Character	Key LARC^b^ method in story	Other content in the story
Moving to the city to escape a violent husband and starting in sex work	Ciku	Implant	Intimate partner violence, inconsistent protection, pregnancy, and STI scares
Main partner (husband or boyfriend) resisting the use of condoms and other contraception	Sandra	Implant	Part-time sex work, STI transmission from boyfriend, condom negotiation with boyfriend, contraceptive pill
Pressure to drink alcohol before sex with a client, and resulting adverse consequences	Lynette	IUD^c^	Sexual risk-taking while intoxicated, strategies for reducing drinking.
Experiencing unintended pregnancy, concern about side effects preventing the use of contraception	Olivia	IUD	Unintended pregnancy and fetal loss, rumors about different contraceptive methods
Being arrested and unable to access emergency contraception	Mimi	IUD	Summary of different contraceptive methods from friends and peer educator
Difficulty negotiating condom use with a client, and making assumptions about his STI^d^ or HIV status	Joslyn	Implant	Dual method use with clients, STI myths

^a^FSW: female sex worker.

^b^LARC: long-acting reversible contraceptive.

^c^IUD: intrauterine device.

^d^STI: sexually transmitted infection.

#### On-Demand Messages

Previous research has indicated that messages about reproductive health that are accessible at any time via an on-demand menu are appealing and motivational to women in East Africa [33,37]. Furthermore, interventions that are bidirectional (involving both push and pull components) may have a greater impact than unidirectional messaging [26,44]. The WHISPER on-demand menu was designed to be accessed by participants sending a code via SMS at any time. They could then obtain more information about contraceptive methods and available health services in their area. On-demand messages, along with the linking and functionality of the code menu, were tested during interviews. Example on-demand messages relating to contraceptive implants are provided in Table 3.

**Table 3:** Example message providing a link to on-demand content, SMS codes, and corresponding on-demand messages.

Push message linking to on-demand menu^a^ and code to trigger message (sent via SMS)		Corresponding on-demand message^a^
**WHISPER Alert: Mimi found out that the implant protects you from pregnancy for 3 to 5 years! Want to know more about the implant? Reply 11 to this text**		
	11	Implants are small rods placed under the skin of a woman’s arm. Highly effective for 3 to 5 years. It can be removed anytime. For married and singles. May cause light irregular bleeding. When removed, you can become pregnant with no delay. No infertility or birth defects. WHISPER main menu, reply 00. For more information, reply 12.
	12	Implants: Benefits 13, Side effects 14, Bleeding side effects 15, True facts 16, Insertion/Removal 17, WHISPER main menu 00.
	15	Implant bleeding side effects: Monthly bleeding may be lighter and irregular during the first year, then lighter, more regular, and infrequent. Monthly bleeding may stop for some women. If bleeding stops it is not harmful—blood does not build up inside the womb.

^a^Example messages contain final content, including any modifications made during phase 2.

### Phase 2: Testing and Refining Messages

The second phase involved testing and refining draft messages based on detailed feedback from FSWs in 6 workshops and 12 interviews. A semistructured workshop guide covered responses to specific messages, overall feedback on the program, and preferences for program structure, timing, delivery, and vernacular. A female researcher who had experience working with FSWs was employed to moderate the workshops rather than an FSW peer educator because this provided more anonymity and privacy to participants. Also, experienced facilitating groups was considered necessary to cover content efficiently but in a sensitive way. A note-taker was also present.

Workshop participants were recruited through purposive sampling of sex work venues (*hotspots*) by FSW peer educators. Peer educators with connections to different hotspots across two subcounties of Mombasa (Kisauni and Changamwe) were selected to be recruiters. Recruiters sought participants with a range of ages and education levels. To be eligible for the workshops, women had to be at least 16 years old, have received money or goods in exchange for sex in the previous 6 months, self-report that they were not currently pregnant or planning a pregnancy in the next year, and own and use a mobile phone. These criteria were consistent with those in the subsequent RCT [29].

We modified the intervention based on the results of the workshops and tested it in 12 one-on-one interviews with participants who met the same eligibility criteria. Interviews tested the technical performance of the SMS system, reactions to the use of on-demand (*pull*) messages, and interpretation of specific messages where there was uncertainty about meaning.

Workshops and interviews were audio-recorded, and detailed notes taken during the sessions were augmented with data from the recordings and translated into English, where necessary, by research assistants. A mixed deductive and inductive thematic approach to analysis was adopted [45]. A list of predetermined codes was used to obtain specific information about message delivery, wording, and preferred content domains. Other codes emerged from the data and were analyzed thematically, particularly in relation to how FSWs responded to the content of different messages and related this to their own experiences, and how behavior change strategies employed by the messages resonated with participants. We analyzed the data using NVivo 11 (QSR International Pty Ltd).

Participants of both workshops and interviews provided written consent before proceeding. They were provided with refreshments and given 500 Kenyan shillings (approximately US $5) to reimburse them for their time and travel costs. The study was approved by the Monash University Human Research Ethics Committee (Australia) and the University of Nairobi and Kenyatta Hospital Ethics Committee (Kenya).

Following data analysis, we refined the intervention further by making recommended wording changes, emphasizing certain content, finalizing the structure (order, timing, and frequency of messages), and resolving technical implementation issues.

## Results

### Workshop Participant Characteristics

We held 6 workshops, each with 7 FSWs, in November 2015 to test the draft messages and refine the intervention. Workshops A and E were held solely with women who had experienced unintended pregnancy to allow open discussion of this issue. Most participants were in their mid-20s (median age 24 years, IQR 20-30) with some secondary education (secondary: 20/42, 48%; primary 15/42, 36%; tertiary: 7/42, 17%) and at least 1 child (34/42, 81%). They worked from a range of hotspots, with half working from bars or nightclubs. Just over half of participants owned a smartphone (the remainder had feature or basic mobile phones), and almost all used SMS at least daily. It was common for participants to share text messages (35/42, 83%), mostly with friends, and some also with family, boyfriends, and clients.

### Responses to Message Content

#### Importance of Topics and Relevance

Participants felt that the topics covered were high priorities for FSWs and would be useful to their community. They confirmed that unintended pregnancy was an important issue that caused fear and stress, and relayed personal experiences of getting pregnant unintentionally. They particularly liked messages that gave general pregnancy prevention advice and information about IUDs.

Many sex workers fear getting pregnant [more] than HIV.

Age 19, Kisauni, workshop F

Message 2 is important…as female sex workers we must use family planning because we have many clients and we need to protect ourselves from becoming pregnant.

Age 20, Kisauni, workshop E

There was a strong positive response to messages on rights, violence, and alcohol use, particularly when violence hotlines were provided, and practical tips were given to reduce alcohol-related harms:

Many sex workers do not know their rights so by sharing with them [these] messages they will be informed.

Age 24, Changamwe, workshop D

Messages were considered highly relevant and spoke to participants’ real experiences, particularly the role model stories, with which participants strongly identified.

This information talks about what sex workers go through.

Age 19, Changamwe, workshop F

Many women volunteered personal stories that echoed message content. Common scenarios were pregnancy scares, difficulty negotiating condom use, experiences of violence, and contraceptive side effects. Role model stories in which the character gets drunk and then needs to use emergency contraception, and in which a woman overcomes contraceptive myths to use an IUD prompted the most discussion and personal anecdotes.

It is realistic. I had the experience when the condom busted and I was unable to access e-pills on time, therefore I conceived a baby and I had no option of aborting, therefore I carried pregnancy to term.

Age 35, Kisauni, workshop E

This thing happens to sex workers and it has happened to me, too.

Referring to unprotected sex while drunk; age 36, Kisauni, workshop C

#### Appeal and Tone

Most women found the messages interesting and appealing, and several commented that the messages stimulated an interest in them to find out more. The majority in all groups agreed that they felt inspired by the role model stories.

It is inspirational especially when Sandra [character in a story] visits a health center for screening and also consults friends on STI prevention.

Age 40, Changamwe, workshop B

I am inspired. It shows us different family planning methods for example depo, IUD.

Age unknown, Kisauni, workshop E

Positive tone also contributed to the appeal. When specifically asked about tone, the most common responses were that the messages were friendly (mentioned 21 times), educational (16), and polite (9). Six women also commented spontaneously that the messages were caring:

They are friendly because they let you know that there is someone who cares for you.

Age 44, Changamwe, workshop B

It is friendly. The message is like peers talking to me. It is not official.

Age 35, Kisauni, workshop E

Participants were asked if FSWs would trust the information provided. Most agreed they would, because the messages were caring and relevant to sex workers, and their community had been involved in developing them.

They will trust [the information] because somebody is caring for them.

Age 22, Changamwe, workshop F

This information is good and they will accept it and also [because] we have been involved.

Age 24, Changamwe, workshop B

### Responses to Behavior Change Strategies

Behavior change strategies adopted from social cognitive theory that were used to develop messages resonated with women. Strategies that were most strongly echoed in their responses were the provision of knowledge, change in outcome expectations, self-efficacy and skill development, and empowerment.

#### Knowledge Gain

A large number of participants reflected that the messages taught them new and useful information. This was the response to both the program overall and specific topics, particularly messages on contraceptive options and side effects, IUDs, condoms, HIV, and alcohol. Participants from workshop A, who were less educated than other groups, were particularly keen to learn more.

I would like to learn more so I would enroll [in the program].

Age 23, Changamwe, workshop A

Friends would want to know more…Yes I will be taught then share the information, especially among sex workers on unwanted pregnancies.

Age 22, Kisauni, workshop D

Specific knowledge gaps were identified as negatively affecting individual participants and their community. Knowledge gaps in HIV transmission were mentioned 4 times, condom usage techniques 3 times, side effects of family planning twice, appropriateness of using IUD with multiple partners twice, menstrual cycles twice, and alcohol and rights once each.

Women frequently mentioned how messages challenged prevalent myths about contraception, particularly about side effects and appropriate use of IUDs:

I did not know that one can use a coil [IUD] and still have many partners.

Age 23, Changamwe, workshop A

I can relate to this episode because I knew with sperms my sitting allowance [buttocks] would increase and my side mirror [hips] would expand, but that was a myth. I have learnt.

Referring to myth that sperm in the vagina is beneficial; age 19, Kisauni, workshop F

However, some described incorrect ideas that they or their peers still held about contraception:

The coil is not good for sex workers because of the nature of work. We have different men of different [penis] sizes.

Age 38, Kisauni, workshop E

Only 2 participants stated that they did not learn anything new, indicating that the level of information was generally well targeted to participants’ background knowledge.

#### Outcome Expectations

*Outcome expectations* refers to the beliefs one holds about the outcomes that will result from a specific behavior [42]. Many messages triggered participants to think about the outcomes of their behaviors, both positive and negative, particularly in relation to family planning. They were also prompted to think about the outcomes of heavy alcohol use, STI prevention, and service utilization. Examples of the outcomes they reflected on are presented in Table 4.

**Table 4:** Outcome expectations raised by workshop participants and corresponding quotes.

Outcome expectations	Example quotes
An outcome of using family planning is not getting pregnant and hence avoiding related stress	“If I am with a client and I am on family planning, I will not fear issues of pregnancy.” (Age 24, Changamwe, workshop B) “When she [character in a role model story] uses coil, she is free and does not have fear of getting pregnant.” (Age 23, Kisauni, workshop D)
Some contraceptive methods cause negative outcomes in the form of side effects but these are less severe than many perceive and can be addressed	“These contraceptives have different effects: they lie to the body that you are pregnant [due to amenorrhea], but if you know the effects there is need not to worry.” (Age 20, Kisauni, workshop D) “It has inspired me, because I can use a coil and when I want a baby I can return to fertility and conceive.” (Age 19, Kisauni, workshop E)
Getting drunk results in increased risk and bad business	“If I get drunk when I go to the hotspot I will not be able to negotiate well with the client and I might be violated. I will not be able to get what I wanted.” (Age 19, Changamwe, workshop F) “When I am sober I will take care of myself from drama and keep myself safe, as sometimes men take advantage if one is drunk; he may refuse to pay you, steal your money and phone, or even not use a condom.” (Age 36, Kisauni, workshop C)
If one accesses a service, they can expect to be provided with good quality care	“When I go the clinic I can get help for an implant or STI treatment.” (Age 20, Changamwe, workshop A) “I have learnt that a health worker can listen to a sex worker and give advice.” (Age 24, Changamwe, workshop D)

Role model stories appeared particularly well suited to supporting changes in outcome expectations and triggered responses in which women reflected on the behaviors of the characters, and the outcomes of their own behaviors and those of peers:

Yes; Lynette [character in story] was drunk and did not have a family planning method, if she had coil the situation could have been avoided.

Age 30, Kisauni, workshop C

My friend had a fear of using a coil, but when she went to hospital she was given advice and more information and she ended up using it, and it is not disturbing her.

Age 30, Kisauni, workshop E

#### Self-Efficacy, Skills, and Action

Participants’ comments indicated a belief that they or their peers are capable of adopting certain behaviors, demonstrating self-efficacy for healthy behavior. They also reflected that some of the messages improved their skills and confidence to adopt new behaviors. Messages that provided specific skills and techniques to lower drinking risk, and specific tips on condom use, were particularly well-received:

I can talk to the waiter and exchange beer with water.

Age 26, Kisauni, workshop C

Violence and rights messages prompted statements reflecting increased self-efficacy for recognizing rights, seeking help, and negotiating with clients:

I know now I am the boss and I can negotiate for payment with clients.

Age 22, Kisauni, workshop F

These messages will teach them their rights, and how they can negotiate and report cases if violated.

Age 18, Kisauni, workshop F

Messages on what to expect from service providers also prompted a response that suggested women felt capable of accessing services:

It is true—the job of the health care is to give services, and I can find a clinic where I am comfortable.

Age 26, Kisauni, workshop C

Participants liked messages that suggested specific actions or plans, and they were triggered to think about what they should do in different situations and how they could make the best use of the messages:

I will put a reminder on the message that I have received, for example when I am at the hotspot.

Age 19, Kisauni, workshop F

If one contraceptive is not good for you, change to another one.

Age 22, Kisauni, workshop D

When I go out I should have a friend or talk to the receptionist at the hotspot to check on my security, and not go with the money in the room.

Age 30, Kisauni, workshop C

#### Empowerment

Empowerment refers to a process in which individuals and communities gain control over their lives and the issues that most affect them, and includes the development of self-confidence and self-reliance [46-48]. The responses of women indicate that they found the messages empowering to both themselves and their community, particularly messages about violence and rights:

It is about me, myself and I. I deserve to be happy and know my rights. Yes I like this message [about rights of sex workers].

Age 24, Changamwe, workshop D

We should visit people who can listen to our voice or our complaints, and health workers should not stigmatize us when we go for services.

Age 23, Kisauni, workshop D

There was a sense that the messages prompted improved morale and inspired them to take action. A number of women specifically mentioned the importance of being in control, particularly in response to role model stories. Stories about the use of LARCs also prompted a sense of being free from the fear of getting pregnant:

They give me morale to use condoms.

Age 31, Changamwe, workshop A

Dual methods remove fear. I have total control when I have the implant and use condoms.

Age 23, Changamwe, workshop A

Participants were overwhelmingly in favor of sharing the messages with other sex workers and friends, and to a lesser extent, with family members, boyfriends, clients, and health workers. Almost all said they would share messages when asked directly, and many said that they would do so without prompting, consistent with the existing practice of frequent sharing. The desire to share influenced their preferences for message delivery. Participants in workshop E preferred SMS because it is an easy format to share. Those in workshops A and B wanted to receive the messages before starting work, to allow time to discuss them with others at the hotspot. Many indicated that it was important for both sex workers and the broader community to have access to this information, and that, as holders of the messages, they would be empowered to provide it. There was a real enthusiasm expressed for teaching others:

My friends do not have this information, therefore I will reach out to them and share with them.

Age 31, Changamwe, workshop A

I will share with 15 and 16 year age groups, because they do not know about family planning and they are already engaging in sex.

Age 20, Kisauni, workshop E

I will share with my clients so that they can reach their spouses.

Age 33, Kisauni, workshop E

By teaching others these messages they will help me to remember.

Age 16, Changamwe, workshop F

### Risks of the Program

One workshop participant thought that she could contact WHISPER to receive emergency assistance (“If am assaulted I can send message or call to get help”; age 40, Kisauni, workshop C). As WHISPER is an automated system, such requests cannot be followed up, and it was concerning that the women may have thought they could depend on the program in this way. This was addressed in subsequent changes to the program (described below).

Breach of privacy was also raised as a potential risk. Participants in 3 workshops discussed the risk that someone else would see the messages and would assume that they were sex workers and/or HIV positive. Some were afraid that this could cause conflict with their boyfriends.

It will bring conflict between me and my boyfriend who might be nosy especially on information on STIs.

Age 24, Changamwe, workshop B

It depends on the person and the relationship you have with them. For example, if a parent sees information about a condom he or she will react, but you can explain. If a client or a boyfriend sees information on HIV he will panic.

Age 19, Kisauni, workshop F

However, not all agreed, and there was a discussion about how the messages might be good for other people, including their boyfriends, illustrated in this interaction:

Even the boyfriends want to plan a family, so they cannot deter us from using this service.

Age 35, Changamwe, workshop B

These messages will be good for both parties—man and woman.

Age 40, Changamwe, workshop B

Others felt that the messages would be socially acceptable. For example, workshop A participants thought friends and health care workers would be impressed that they were *careful with their lives*:

My boyfriend, family or friend will say I am informed.

Age 23, Changamwe, workshop A

Another risk is that the program would not overcome barriers to healthy behaviors in sex work. Responses illustrated how some barriers cannot be overcome by an individually targeted intervention alone. For example, a role model story about a client offering to pay extra for no condom prompted discussion in workshop F about the need to balance conflicting outcome expectations of different courses of action. This reflected sex workers’ need to continually assess risk, and the fact that money and immediate safety are often higher priorities than pregnancy and STI prevention.

The client of Joslyn [character in the story] in this case was polite, because he said he will call next week, but most clients will become abusive if you refuse to not use condoms.

Age 19, Changamwe, workshop F

The issue is money. That is why female sex workers risk going without a condom—so that she might get a client.

Age 19, Kisauni, workshop F

I had a friend who had the same issue. She judged the guy with looks because the guy had money. She did not negotiate for condom before. The money was huge. The lady refused because this guy insisted no condom.

Age 22, Kisauni, workshop F

### Response to On-Demand Messages

Interview participants were sent messages with a link to the on-demand system. In all, 7 of 12 participants found it *very easy* to access messages on demand. Others had minor difficulties, and 2 had genuine difficulty and had to be directed by the interviewer. These women had lower education than other participants.

Many women liked having the option to retrieve more information and the interactive aspect of the system. They talked about the ease of getting detailed information on their phones rather than having to seek it out from health professionals, and the ability to refer back to such messages later. A number of women did not feel the initial message on a topic contained new information, but obtaining more detail allowed them to gain a greater understanding.

It has motivated me since I can get instant replies and can be helped instantly.

Age 26, Changamwe

It is like revision [on] family planning⸺when I am reading I am being enlightened more and remembering, it is easy.

Age 33, Changamwe

Messages about health services were considered very useful to participants and their peers as they provided information that was not easy to obtain and saved the time and resources needed to find appropriate services. The emergency message (developed in response to workshop feedback—described below) was particularly popular and seen as important.

When I need help, or having an emergency, they have provided a number which I can call for free in case of violence and has given me a whisper menu too. In short they have not left me hanging from the situation I may be experiencing.

Age 30, Kisauni

There were some technical issues during interviews, including delayed receipt of messages and (erroneous) warnings received from network providers, which deterred some women from continuing. Despite these challenges, most interview participants were very engaged in the process of retrieving pull messages, and those who had initial difficulty still enjoyed the process. When asked directly, all agreed that they would like to continue using the system.

### Intervention Structure and Final Delivery

#### Intervention Delivery Preferences

Workshop participants had generally consistent preferences regarding how the intervention should be delivered. The majority were in favor of text rather than voice modality and preferred push messages to retrieving content via a pull system. Most women reported that their texting practices involved a mixture of English and Swahili, and they favored English for health messages, with some keywords or phrases in Swahili. Participants wanted to receive messages several times a week for at least 1 year and preferred to receive them in the late morning on set days to align with their typical work schedules.

#### Refinement of the Intervention

A number of changes were made to the intervention content and form based on findings. To minimize the risk of women expecting emergency assistance from WHISPER, a message was included on what to do in an emergency, specifically around violence. An *error* message was also developed that was triggered if they tried to send content other than the prespecified codes. These were well received on testing in the interviews.

The other key concern identified was the risk of sex work status being discovered by clients or boyfriends viewing the messages. In response to this, we minimized overt references to sex work and clients wherever possible.

Suggestions were adopted from participants regarding the use of specific words and terms, in both Swahili and English. Terms of endearment like *mrembo* (beautiful) and *darling* were incorporated into the messages, and *family planning* was adopted consistently as FSW’s preferred term for pregnancy prevention.

A number of strategies were adopted to address the technical challenges encountered using the on-demand system. These included testing the system with each participant during their enrolment and incorporating introductory messages that explained how to use the on-demand menu.

#### Final Intervention Schedule

The intervention components and delivery schedule were finalized based on workshop and interview results. Over a 12-month intervention period, participants received SMS 2 to 3 times per week, alternating push messages with role model stories every month. A total of 82 push messages were developed for the intervention (see examples in Table 1). In addition, 7 reminders for study visits and 19 alerts linking to the pull system (Table 2) were sent to participants. Six role model stories were sent, each with 4 or 5 episodes (Table 1). Messages were scheduled for mornings on set days, in line with participant preferences.

## Discussion

We provide the first description of the development of a digital health intervention for FSWs that uses a comprehensive SRHR framework. The participatory approach enabled FSWs to influence the range and content of topics included in the intervention [49] and to enhance the relevance and salience of messages, and the participants themselves confirmed that their involvement improved the perceived trustworthiness of the messages. The benefits of a co-design approach have been observed in other mHealth studies [32]. Co-design is critical for handling sensitive content matter that may be interpreted differently by different communities [50].

Furthermore, health behavior change interventions are more effective when they are based on social and behavioral science theory, and the use of multiple theories may increase intervention effectiveness [51,52]. WHISPER utilizes multiple theories to guide the intervention framework and specify intermediate behavior change outcomes [42,43]. Notably, the adopted strategies from Bandura’s social cognitive theory [35] were frequently highlighted by workshop participants, confirming the applicability and utility of theory for guiding intervention design.

The messages increased participants’ feelings of empowerment [46-48] and social support [43]. There was a strong sense of being part of a community; many women reflected on how messages would help their friends, or how they could share the knowledge they had gained, rather than focusing solely on how it would help them as individuals. These findings suggest that WHISPER may capitalize on and enhance community cohesion. Social cohesion has been linked to safer sex behaviors [53] and is important for the success of community empowerment interventions, which may otherwise be undermined by mistrust and competition among FSWs for scarce resources [7]. The desire to share messages with peers and the broader community suggests that social diffusion is also likely to contribute to the effectiveness of the intervention [43].

Participants reinforced the importance of the selected SRHR topics and confirmed that unintended pregnancy is a major concern for sex workers. The team was careful to ensure that scenarios were not overly optimistic and appropriately represented known barriers. Content addressing family planning myths was stated in different ways and different formats (push and pull messages and role model stories) to maximize the potential that participants would engage with and learn from the WHISPER content so that myths would no longer represent barriers to participants.

In addition to family planning, alcohol use and gender-based violence were viewed as important. Strategies for reducing drinking provided in the text messages were adapted from effective harm reduction interventions [54], and this practical emphasis resonated strongly with participants. Experiences of violence were frequently described and noted as a barrier to adopting safer sexual practices. Although an individual health promotion intervention cannot address the structural causes of violence or change the behavior of perpetrators, participant responses indicate that messages about violence improved knowledge of rights, were empowering, and provided much-needed advice about how to reduce risks and access services.

The intervention was highly acceptable to both workshop and interview participants. Women were interested and engaged in both the content and the format of delivery, with role model stories eliciting particularly enthusiastic discussion, and SMS confirmed as the preferred technology. Workshop and interview participants demonstrated familiarity and comfort using SMS, and desire to learn more, suggesting that it is feasible for SRHR messages to be sent regularly over a year to this population. Testing during interviews confirmed the feasibility of the on-demand system. Most participants could retrieve pull messages with relative ease; however, women who are less educated or have less experience with mobile phones may experience difficulty using this system.

There were some technical issues, including a network warning that could not be deactivated. Similar problems have been identified by other implementers of mHealth programs [55,56], highlighting the importance of real-time testing and the need to consider how to overcome aspects of mobile platforms designed for commercial rather than public health applications.

We have demonstrated that WHISPER is feasible to implement and acceptable to the target audience; however, this may not translate to sufficient participant engagement to produce better health outcomes. Engagement with a digital health program incorporates not only the subjective and cognitive responses that are triggered (which are explored in this paper) but also the extent of use [57] (eg, the number and frequency of messages received), which will be measured during the trial. The evaluation of engagement in digital health interventions has not been well characterized and is an important area for further research [57].

Our research revealed several risks to participation in a digital health SRHR intervention. First, participants believed that they could receive emergency assistance from WHISPER. It is possible that the friendly and personal tone—while effective in generating intervention engagement [58]—creates an expectation that participants are interacting with real people rather than an automated system. Revisions were made to minimize this risk.

Second, disclosure of sensitive messages could result in increased conflict with boyfriends or clients, although it also has the potential to improve communication with partners. Disclosure risk has been explored during the development of mHealth interventions for HIV [58-60], and an increase in intimate partner violence was an unintended consequence of a contraceptive mHealth program in Bangladesh [61]. However, few studies report on the potential harms from women’s participation in SRHR digital health programs, and this is an important area for further research [61,62].

This study had some important limitations. We used purposive sampling and cannot ensure that workshop participants were representative of the larger FSW population. In addition, our approach to data collection and analysis was highly directive, and some messages were not tested because they were from preexisting mHealth interventions [37]. This approach to data collection yielded the specific information needed to develop the intervention, but it was not designed to reach data saturation, and it is possible that some critical feedback was not obtained.

This research provides a clear illustration of the many issues that preoccupy FSWs in their day-to-day lives—beyond the traditional biomedical focus on HIV risk and transmission. Our results support the need for health promotion interventions that utilize a participatory approach to intervention development and are based on social and behavioral science to increase their relevance and effectiveness. The resulting WHISPER digital health intervention reflects the complex reality of FSWs’ daily lives and provides a feasible, engaging, and confidential approach for addressing their SRHR concerns and needs.

## Abbreviations

FSW: female sex worker
IUD: intrauterine device
LARC: long-acting reversible contraceptive
NHMRC: National Health and Medical Research Council Australia
RCT: randomized controlled trial
SRHR: sexual and reproductive health and rights
STI: sexually transmitted infection
WHISPER: Women’s Health Intervention using SMS for Preventing Pregnancy
